# Poly (ADP-Ribose) Polymerase Mediates Diabetes-Induced Retinal Neuropathy

**DOI:** 10.1155/2013/510451

**Published:** 2013-11-21

**Authors:** Ghulam Mohammad, Mohammad Mairaj Siddiquei, Ahmed M. Abu El-Asrar

**Affiliations:** Department of Ophthalmology, College of Medicine, King Saud University, P.O. Box 245, Riyadh 11411, Saudi Arabia

## Abstract

Retinal neuropathy is an early event in the development of diabetic retinopathy. One of the potential enzymes that are activated by oxidative stress in the diabetic retina is poly (ADP-ribose) polymerase (PARP). We investigated the effect of the PARP inhibitor 1,5-isoquinolinediol on the expression of the neurodegeneration mediators and markers in the retinas of diabetic rats. After two weeks of streptozotocin-induced diabetes, rats were treated with 1,5-isoquinolinediol (3 mg/kg/day). After 4 weeks of diabetes, the retinas were harvested and the levels of reactive oxygen species (ROS) were determined fluorometrically and the expressions of PARP, phosporylated-ERK_1/2_, BDNF, synaptophysin, glutamine synthetase (GS), and caspase-3 were determined by Western blot analysis. Retinal levels of ROS, PARP-1/2, phosphorylated ERK_1/2_, and cleaved caspase-3 were significantly increased, whereas the expressions of BDNF synaptophysin and GS were significantly decreased in the retinas of diabetic rats, compared to nondiabetic rats. Administration of 1,5-isoquinolinediol did not affect the metabolic status of the diabetic rats, but it significantly attenuated diabetes-induced upregulation of PARP, ROS, ERK_1/2_ phosphorylation, and cleaved caspase-3 and downregulation of BDNF, synaptophysin, and GS. These findings suggest a beneficial effect of the PARP inhibitor in increasing neurotrophic support and ameliorating early retinal neuropathy induced by diabetes.

## 1. Introduction

Diabetes is a metabolic disorder characterized by hyperglycemia and often leads to numerous microvascular complications, including retinopathy. Diabetic retinopathy (DR) is a multifactorial disease, and persistent hyperglycemia appears to be a major contributor to its development. Recent evidence suggests that diabetic retinopathy is a progressive neurodegenerative disease as evidenced by the presence of apoptotic cells in all retinal layers. Activation of caspase-3 is part of the mechanism of apoptosis. Visual dysfunction is initiated early after the onset of diabetes and progresses independently of the vascular lesions [1–4]. The exact molecular mechanisms which contribute to development of diabetes-induced retinal neuropathy remain largely unknown. Reactive oxygen species (ROS) production is increased in the retina in diabetes, and it is considered as one of the major contributors of retinal metabolic abnormalities postulated to be involved in the development of diabetic retinopathy. Administration of antioxidants to diabetic rats protects the retina from oxidative damage, and also the development of retinopathy [5–8]. In diabetes, retinal neuropathy is associated with enhanced oxidative stress resulting from excess generation of ROS that often leads to retinal microvascular cell death [3, 9, 10].

Enhanced ROS level causes reduced levels of brain-derived neurotrophic factor (BDNF), a protein belonging to the neurotrophin family. BDNF is expressed in retinal ganglion cells (RGCs) and Müller cells [11] and is important for the survival of retinal ganglion cells [12]. BDNF is important for neural development and cell survival and is essential for molecular mechanisms of synaptic activity [13]. Recent studies suggested that the early retinal neuropathy of diabetes involves the reduced expression of BDNF and can be ameliorated by an exogenous supply of this neurotrophin [1, 3]. ROS also decreases the level of synaptophysin, a synaptic vesicle protein for neurotransmitter release which is widely expressed in the retina [14, 15]. Glutamate, the excitatory neurotransmitter in the retina, is released by photoreceptors, bipolar cells, and ganglion cells and mediates the transfer of visual signals from the retina to the brain [16]. Excess glutamate release in hypoxic-ischemic conditions causes excitotoxic damage to the RGCs through activation of ionotropic and metabotropic glutamate receptors. The synaptically released glutamate is taken up by Müller cells where glutamine synthetase converts it into glutamine. Several studies found that the expression of GS was significantly decreased in the diabetic rat retinas [17, 18]. These dysfunctions resulted in elevated glutamate levels in the diabetic retinas [17, 19, 20], which might induce retinal neurodegeneration via glutamate excitotoxicity. Synaptophysin protein is decreased in the retina of the streptozotocin (STZ)-induced diabetes model through the ROS-extracellular signal-regulated kinase 1 and 2 (ERK_1/2_) suggesting the involvement of cross talk between mitogen-activated protein kinases (MAPK) pathway signals and neurodegeneration in the diabetic retina [3, 21]. It was also demonstrated that the reduction of BDNF and synaptophysin in the diabetic retina was attenuated by the antioxidant lutein, indicating that this change was partly caused by excessive oxidative stress [3]. One of the major consequences of oxidative stress is DNA damage. High level of ROS induces DNA strand breaks in the retina by hyperglycemia [22], and ROS-induced DNA single-strand breakages were considered an obligatory step for Poly(ADP-ribose) polymerase (PARP) cleavage/activation. 

PARP is a nuclear enzyme that regulates several cellular events including DNA repair, cellular division and differentiation, DNA replication, transformation, gene expression and amplification, mitochondrial function, and cell death. Altered activity of PARP is reported under many pathological conditions, including diabetes. Extensive experimental data generated in both tissue culture and animal models signify that diabetes-induced PARP activation or its overexpression in the retina by DNA damage induces cell death; a phenomenon that precedes the development of histopathologic change [22–25]. Recently, it was documented that PARP activation contributes to superoxide anion radical and peroxynitrite formation in peripheral nerve, vasa nervorum, and aorta of STZ-induced diabetic rats and high-glucose exposed human Schwann cells [26]. Furthermore, PARP inhibition counteracted diabetes-induced systemic oxidative stress and 4-hydroxynonenal adduct accumulation in peripheral nerves and improved nerve fiber function [27]. These findings suggest that PARP activation in diabetes is bi- rather than unidirectional. However, so far no study has shown the cross talk between PARP activation and retinal neuropathy. Therefore, in this study, we explored the hypothesis that PARP activation mediates retinal neuropathy by reducing BDNF, synaptophysin, and GS levels in the diabetic retina. To test this hypothesis, we measured the levels of ROS generation, PARP, phosphorylated ERK_1/2_ (p-ERK_1/2_), BDNF, synaptophysin, GS, and cleaved caspase-3 in the retina of diabetic animals. In addition, we analyzed whether treatment with the PARP inhibitor 1,5-isoquinolinediol suppresses the neurodegenerative changes in the retinas of diabetic rats.

## 2. Materials and Methods

### 2.1. Induction of Diabetes and 1,5-Isoquinolinediol Treatment

All procedures with animals were performed in accordance with the Association for Research in Vision and Ophthalmology (ARVO) statement for use of animals in ophthalmic and vision research and were approved by the institutional animal care and use committee of the College of Pharmacy, King Saud University. Adult male Sprague Dawley rats of 8-9 weeks of age (200–220 g) were overnight fasted and STZ 65 mg/kg in 10 mM sodium citrate buffer, pH 4.5 (Sigma, St. Louis, MO, USA) was injected intraperitoneal. Equal volumes of citrate buffer were injected in nondiabetic animals. Rats were considered diabetic if their blood glucose was greater than 250 mg/dL. Age-matched normal rats served as controls. 

Two weeks later, the diabetic rats were randomly divided into 2 groups matched for body weight and blood glucose: diabetic group without treatment (D, N = 10) and diabetic group given 1,5-isoquinolinediol (3 mg/kg/day, intraperitoneal; Santa Cruz Biotechnology, CA) (Dib + ISO, N = 10). This dose of 1,5-isoquinolinediol was based on previous studies [28]. After 4 weeks of diabetes, the rats were euthanized by an overdose of chloral hydrate, the eyes were removed, and retina was isolated and frozen immediately in liquid nitrogen and stored at −80°C to be analyzed by Western blot analysis or biochemical assay. 

### 2.2. Immunohistochemical Analysis

The C57Bl/6J mice were made diabetic by intraperitoneal injection of Streptozotocin for five consecutive days. Mice with blood glucose above 200 mg/dL, 3 days after the last injection of streptozotocin, were considered as diabetic. Age-matched normal C57Bl/6J mice served as controls. After 12 weeks of diabetes, the mice were euthanized by an overdose of chloral hydrate, the eyes were removed, and were used for immunohistochemistry by incubation in 10% paraformaldehyde for 30 min. Then the eyes were washed with PBS, fixed in optimal cutting temperature compound (OCT), and immediately frozen in liquid nitrogen for sectioning. Cryosections (10 **µ**m) prepared from mouse retina were fixed with 4% paraformaldehyde and blocked with 10% normal goat serum and incubated with rabbit polyclonal anti-PARP antibody overnight (1 : 150, Cat. no. SC-7150, Santa Cruz Biotechnology, Inc., Santa Cruz, CA, USA). After rinsing the slides with PBS, these were incubated with the secondary antibody (anti-rabbit-FITC conjugated) for 1 hour. The slides were rinsed with PBS, mounted with DAPI-containing mounting media (Vector Laboratories), and imaged with an Olympus BX-UCB fluorescent microscope (20x magnification). 

### 2.3. Western Blot Analysis

Retinas were homogenized in a western lysis buffer (30 mM Tris-HCl, pH 7.4, 250 mM Na_3_VO_4_, 5 mM EDTA, 250 mM sucrose, 1% Triton X-100 with Protease inhibitor). The lysate was centrifuged at 14,000 ×g for 10 min at 4°C, and the supernatant was collected. Protein content was assayed by DC protein assay (Bio-Rad Laboratories, Hercules, CA, USA). The tissue lysates containing 40–50 **μ**g protein were separated on 8–15% SDS-polyacrylamide gels and were transferred onto nitrocellulose membranes. The blots were blocked with 5% nonfat milk in TBST (20 mM Tris-HCl, pH 7.6, 136 mM NaCl, and 0.1% Tween-20).

For detection of PARP, p-ERK_1/2_, BDNF, synaptophysin, and cleaved caspase-3, the membrane was incubated overnight at 4°C with mouse monoclonal anti-PARP (1 : 200, Cat. no. ab110915, Abcam, UK), rabbit polyclonal anti-ERK_1/2_ (1 : 300, Cat. no. ab17942, Abcam), mouse monoclonal anti-phosphorylated ERK_1/2_ (1 : 300, Cat. no. ab50011, Abcam), mouse monoclonal anti-BDNF (1 : 500, Cat. no. SC-65513, Santa Cruz Biotechnology), goat polyclonal anti-synaptophysin (1 **μ**g/mL, Cat. no. AF-5555, R&D Systems, Minneapolis, MN, USA), goat polyclonal anti-GS (1 : 500, SC-6640, Santa Cruz Biotechnology), and rabbit monoclonal anti-caspase-3 (1 : 300, Cat. no. MAB835, R&D Systems). After overnight incubation with primary antibodies, the membranes were washed four times with TBS-T (5 min each). For PARP, p-ERK_1/2_, and BDNF, the membrane was incubated at room temperature for 1.5 h with anti-mouse secondary horseradish peroxidase-conjugated antibody (1 : 2000, SC-2005, Santa Cruz Biotechnology), for synaptophysin with anti-goat secondary horseradish peroxidase-conjugated antibody (1 : 2000, SC-2768, Santa Cruz Biotechnology), and for ERK_1/2_ and cleaved caspase-3, with anti-rabbit secondary horseradish peroxidase-conjugated antibody (1 : 2000, SC-2004, Santa Cruz Biotechnology). After incubations with secondary antibodies, membranes were washed four times with TBS-T (5 min each) and the immunoreactivity of bands was visualized on a high-performance chemiluminescence machine (G: Box Chemi-XX8 from Syngene, Synoptic Ltd. Cambridge, UK) by using enhanced chemiluminescence plus Luminol (SC-2048, Santa Cruz Biotechnology) and quantified by densitometric analysis using image processing and analysis in GeneTools (Syngene by Synoptic Ltd. Cambridge, UK). As a control, the blots were stripped and detected with a mouse monoclonal anti-**β**-actin (1 : 2000, SC-47778, Santa Cruz Biotechnology), antibody. All data from the three independent experiments were expressed as a ratio to OD. 

### 2.4. Reactive Oxygen Species Measurements

Reactive Oxygen Species (ROS) generation was measured in retinal tissue homogenates using a 2′,7′-dichlorofluorescein-diacetate (DCHFDA) [29]. DCFHDA, a nonfluorescent dye, is cleaved by esterase activity to yield DCFH, which is subsequently oxidized by a variety of ROS to form dichlorofluorescein (DCF), which is fluorescent. Retinas were homogenized in PBS in presence of protease inhibitor using a glass homogenizer. Samples containing 20 **μ**g proteins diluted in PBS were incubated with 5 **μ**M DCFHDA (Invitrogen, CA, USA) in the dark for 15 min. Fluorescence was measured using a spectraMax Gemini-XPS (Molecular Devices, CA, USA) every 15 min for 1 h with excitation and emission wavelengths of 488 nm and 525 nm. 

### 2.5. Apocynin Treatment

STZ-induced diabetic rats were divided into 2 groups: the rats in group I received normal drinking water without any supplementation, and those in group II received drinking water supplemented with apocynin (15 mg/kg/day in drinking water; Santa Cruz Biotechnology) immediately after establishment of diabetes. This dose of apocynin was based on previous studies [30]. Each group had 8–12 rats. After 4 weeks of diabetes, the rats were euthanized by an overdose of chloral hydrate, the eyes were removed, and retina was isolated and frozen immediately in liquid nitrogen and stored at −80°C to be analyzed by Western blot analysis or biochemical assay. 

### 2.6. Statistical Analysis

Each measurement was made in duplicate, and the assay was repeated three or more times. Data are expressed as mean ± SD and experimental groups were compared using the nonparametric Kruskal-Wallis test followed by the Mann-Whitney test for multiple-group comparison. A *P* value ≤0.05 indicated statistical significance. SPSS version 12.0 was used for the statistical analyses.

## 3. Results

### 3.1. Metabolic Changes Induced by Diabetes

The body weights of the diabetic rats were significantly lower and their blood glucose values were more than fourfold higher compared with age-matched normal control rats (170 ± 22 versus 270 ± 28 g and 453 ± 32 versus 111 ± 12 mg/dL, resp.). 1,5-isoquinolinediol treatments to diabetic rats for 2 weeks did not affect the body weight and blood glucose levels compared to untreated diabetic rats (182 ± 25 versus 170 ± 22 g and 469 ± 36 versus 453 ± 32 mg/dL). 

### 3.2. Effect of Diabetes on Retinal Expression and Activation of PARP

**Figure 1:**
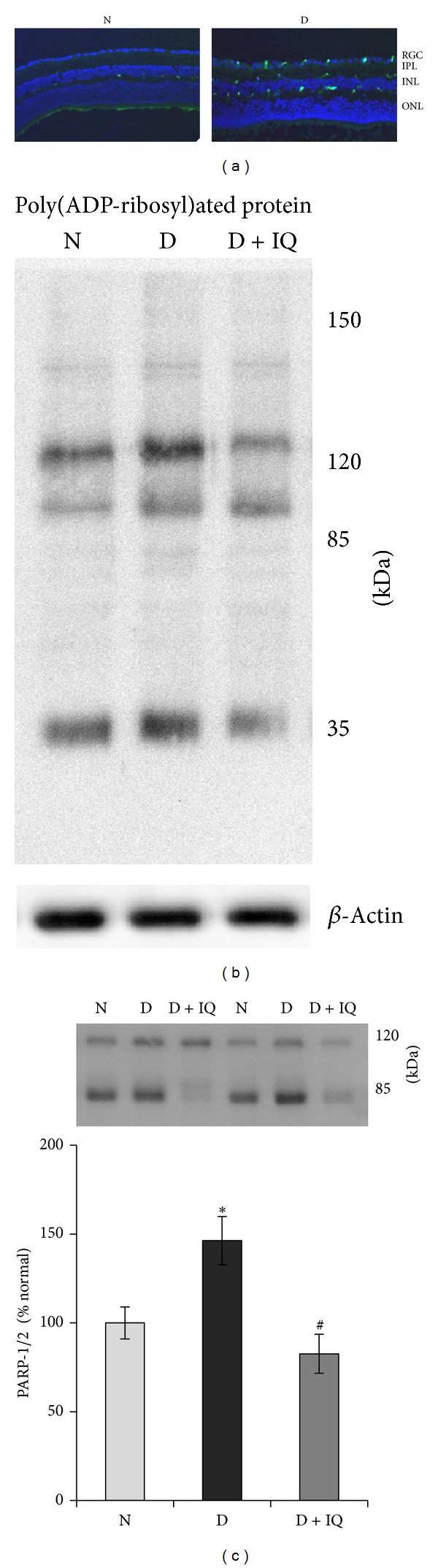
Effect of 1,5-isoquinolinediol on retinal poly(ADP-ribose) polymerase (PARP) expression in diabetes. (a) Cryosections from normal (N) and diabetic (D) retina were subjected to immunostaining using anti-PARP antibodies (green) and DAPI (blue) was used to stain the nuclei. The sections were imaged at 20x magnification using Olympus BX-UCB fluorescent microscope. RGC: retinal ganglion cell; IPL: inner plexiform layer; INL: inner nuclear layer; ONL: outer nuclear layer. (b) Representative Western blot analyses of retinal poly(ADP-ribosyl)ated proteins in control and diabetic rats maintained with and without PARP inhibitor treatment. (c) PARP activation was measured by western blot technique, and the ratio of active PARP-2 (85 KDa) and pro-PARP-1 (120 kDa) was calculated in control and diabetic rats maintained with and without PARP inhibitor treatment. Measurements were made in duplicate in six to eight rats in each group. Western blots are representative of three different experiments. Results are expressed as mean ± SD. Values obtained from nondiabetic rats are considered as 100%. N: nondiabetic rat; D: diabetic rat; D + IQ: diabetic rat treated with 1,5-isoquinolinediol. *P < 0.05 compared with nondiabetic rat. ^#^ *P* < 0.05 compared with diabetic rat.

Retinal PARP immunoreactivity (green FITC fluorescent staining) was upregulated in diabetic rats compared with nondiabetic controls. PARP was localized primarily in the ganglion cell and inner nuclear layers of the retina (Figure 1(a)). Representative Western blot image of retinal poly(ADP-ribosyl)ated proteins in control and diabetic rats was maintained with and without PARP inhibitor treatment (Figure 1(b)). Western blot analysis demonstrated significant upregulation of PARP-1/2 expression in diabetic retinas compared to nondiabetic retinas. The expression of PARP-1/2 protein in the retinas of diabetic rats was upregulated by about 40% as compared to the retinas of nondiabetic rats (Figure 1(c)). 

### 3.3. Effect of Diabetes on Retinal Expression of Mediators and Markers of Neurodegeneration

**Figure 2:**
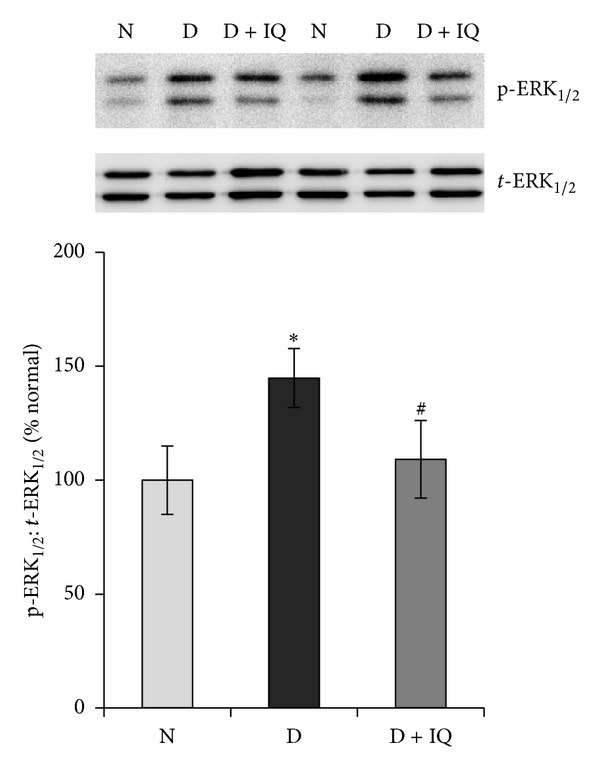
Effect of 1,5-isoquinolinediol on retinal ERK_1/2_ activation in diabetes. Relative abundance of phosphorylated ERK_1/2_ (p-ERK_1/2_) and total ERK_1/2_ (*t*-ERK_1/2_) was determined by Western blotting, followed by densitometry. Data are expressed as percentage change in phosphorylation over *t*-ERK_1/2_ and are expressed as mean ± SD. Values obtained from nondiabetic rats are considered as 100%. N: nondiabetic rat; D: diabetic rat; D + IQ: diabetic rat treated with 1,5-isoquinolinediol. *P < 0.05 compared with nondiabetic rat. ^#^ *P* < 0.05 compared with diabetic rat.

**Figure 3:**
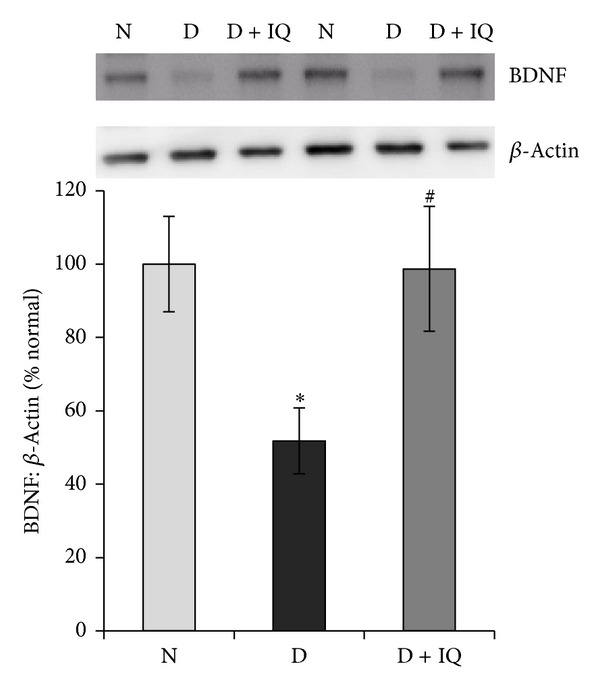
Effect of 1,5-isoquinolinediol on retinal brain-derived neurotrophic factor (BDNF) expression in diabetes. The expression of BDNF in retinal homogenate was determined by Western blotting technique. The histogram represents the mean band intensity (from 6–8 rats in each group) of BDNF adjusted to the intensity of **β**-actin in the same sample. Values obtained from nondiabetic rats are considered as 100%. N: nondiabetic rat; D: diabetic rat; D + IQ: diabetic rat treated with 1,5-isoquinolinediol. *P < 0.05 compared with nondiabetic rat. ^#^ *P* < 0.05 compared with diabetic rat.

**Figure 4:**
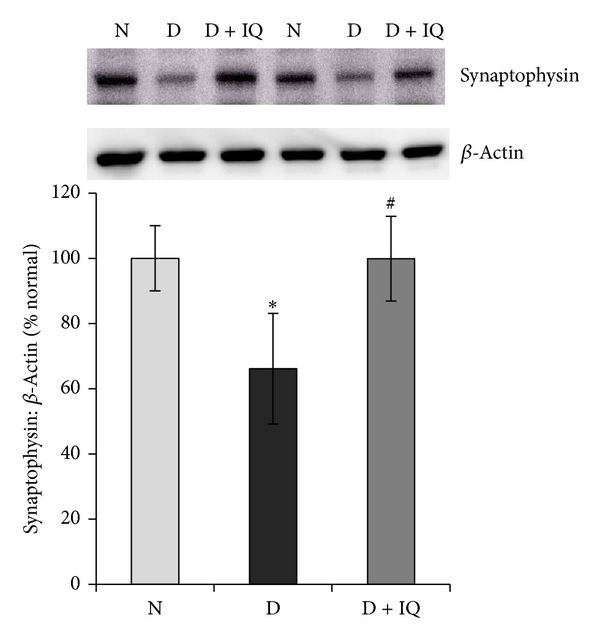
Effect of 1,5-isoquinolinediol on retinal synaptophysin expression in diabetes. The expression of synaptophysin in retinal homogenates was determined by Western blotting technique. The histogram represents the mean band intensity (from 6–8 rats in each group) of synaptophysin adjusted to the intensity of **β**-actin in the same sample. Values obtained from nondiabetic rats are considered as 100%. N: nondiabetic rat; D: diabetic rat; D + IQ: diabetic rat treated with 1,5-isoquinolinediol. *P < 0.05 compared with nondiabetic rat. ^#^ *P* < 0.05 compared with diabetic rat.

**Figure 5:**
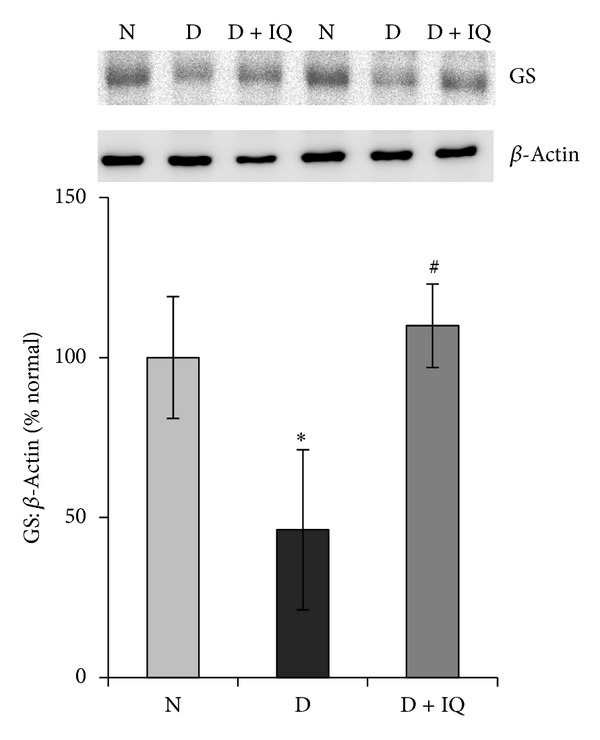
Effect of 1,5-isoquinolinediol on retinal GS expression in diabetes. The expression of GS in retinal homogenates was determined by Western blotting technique. The histogram represents the mean band intensity (from 6–8 rats in each group) of GS adjusted to the intensity of **β**-actin in the same sample. Values obtained from nondiabetic rats are considered as 100%. N: nondiabetic rat; D: diabetic rat; D + IQ: diabetic rat treated with 1,5-isoquinolinediol. *P < 0.05 compared with nondiabetic rat. ^#^ *P* < 0.05 compared with diabetic rat.

**Figure 6:**
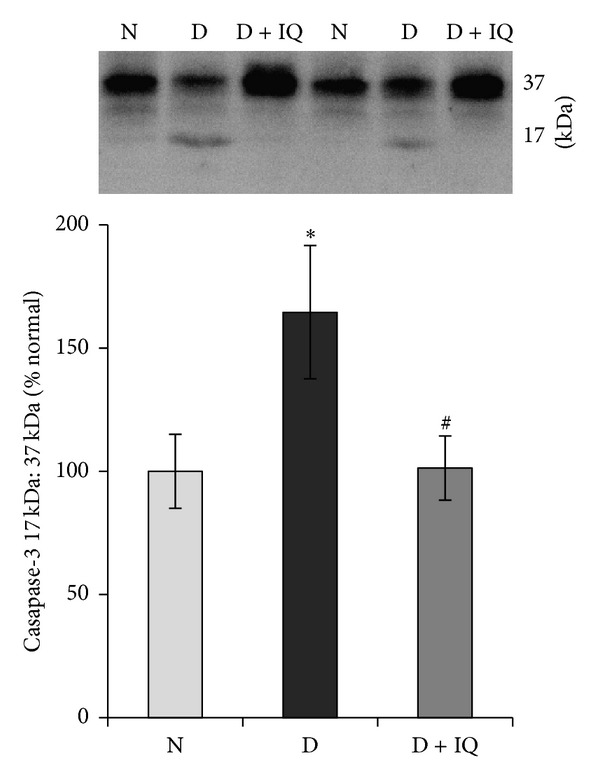
Effect of 1,5-isoquinolinediol on retinal cleavage of caspase-3 in diabetes. Cleavage of caspase-3 was measured by Western blot analysis, and the ratio of active caspase-3 (17 KDa) and pro-caspase-3 (37 kDa) was calculated in control and diabetic rats maintained with and without PARP inhibitor treatment. Measurements were made in duplicate in six to eight rats in each group. Western blots are representative of three different experiments. Results are expressed as mean ± SD. Values obtained from nondiabetic rats are considered as 100%. N: nondiabetic rat; D: diabetic rat; D + IQ: diabetic rat treated with 1,5-isoquinolinediol. *P < 0.05 compared with nondiabetic rat. ^#^ *P* < 0.05 compared with diabetic rat.

Western blot analysis demonstrated significant upregulation of ERK_1/2_ phosphorylation in diabetic retinas compared to nondiabetic retinas. The phosphorylation of ERK_1/2_ protein in the retinas of diabetic rats was upregulated by about 45% as compared to the retinas of nondiabetic rats (Figure 2). The neurotrophin BDNF was significantly downregulated in diabetic retinas compared to nondiabetic controls. BDNF expression in the retinas of diabetic rats was decreased by about 52% compared to nondiabetic controls (Figure 3). The expression of the synaptic vesicle protein synaptophysin in the retinas of diabetic rats was downregulated by about 35% as compared to the retinas of nondiabetic rats (Figure 4). GS is an enzyme which converts glutamate into glutamine; protein expression was significantly decreased by about 58% in diabetic retinas compared to nondiabetic retinas (Figure 5). Cleaved caspase-3, the apoptosis executer enzyme, was significantly upregulated in diabetic retinas compared to nondiabetic controls. Cleaved caspase-3 levels in the retinas of diabetic rats were increased by about 65% compared to nondiabetic controls (Figure 6).

### 3.4. Effect of Diabetes on Retinal ROS Generation

**Figure 7:**
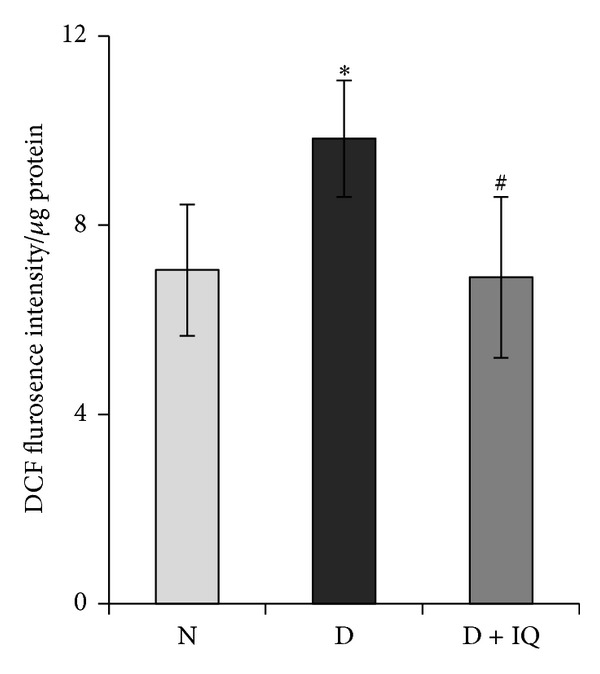
Effect of 1,5-isoquinolinediol on retinal reactive oxygen species (ROS) levels in diabetes. Freshly prepared retinal homogenates were incubated with DCHFDA (5 **μ**M) for 30 min. An equal amount of protein was used to quantitate 2′,7′-dichlorofluorescein fluorescence. N: nondiabetic rat; D: diabetic rat; D + IQ: diabetic rat treated with 1,5-isoquinolinediol. Data are expressed as percent control and mean ± SD from retina from 5-6 rats in each group. *P < 0.05 compared with nondiabetic rat. ^#^ *P* < 0.05 compared with diabetic rat.

Spectrofluorometric analysis demonstrated significant upregulation of ROS generation in diabetic retinas compared to nondiabetic retinas. ROS production in retinas was determined by DCFH-DA probe. The levels of DCF fluorescence intensity in the retina of diabetic rats were increased by 40% as compared to control rats (Figure 7).

### 3.5. The PARP Inhibitor 1,5-Isoquinolinediol Attenuates Diabetes-Induced PARP Upregulation

1,5-isoquinolinediol is a potent PARP antagonist and decreases PARP activity in diabetic retina [28]. We employed 1,5-isoquinolinediol to investigate the antineurodegeneration function in the retinas of diabetic rats. Diabetic rats that were treated with 1,5-isoquinolinediol showed significant attenuation of PARP-1/2 activation by about 60% as compared to untreated diabetic rats (Figure 1(c)).

### 3.6. The PARP Inhibitor 1,5-Isoquinolinediol Attenuates the Effect of Diabetes

Western blot analysis was used to assess the effect of 1,5-isoquinolinediol on diabetes-induced alterations of p-ERK_1/2_, BDNF, synaptophysin, GS, cleaved caspase-3, and ROS. 1,5-isoquinolinediol administration significantly attenuated diabetes-induced upregulation of p-ERK_1/2_ and cleaved caspase-3 by about 40% and 60%, respectively. In the 1,5-isoquinolinediol-treated diabetic rats, the decrease in BDNF, synaptophysin, and GS caused by diabetes was attenuated by about 50%, 34%, and 50%, respectively (Figures 2–6). The levels of ROS generation are attenuated by about 35% in the retina of diabetic rats treated by 1,5-isoquinolinediol as compared to untreated diabetic rats (Figure 7).

### 3.7. Effect of the Specific ROS Inhibitor Apocynin on PARP-1/2 Expression

**Figure 8:**
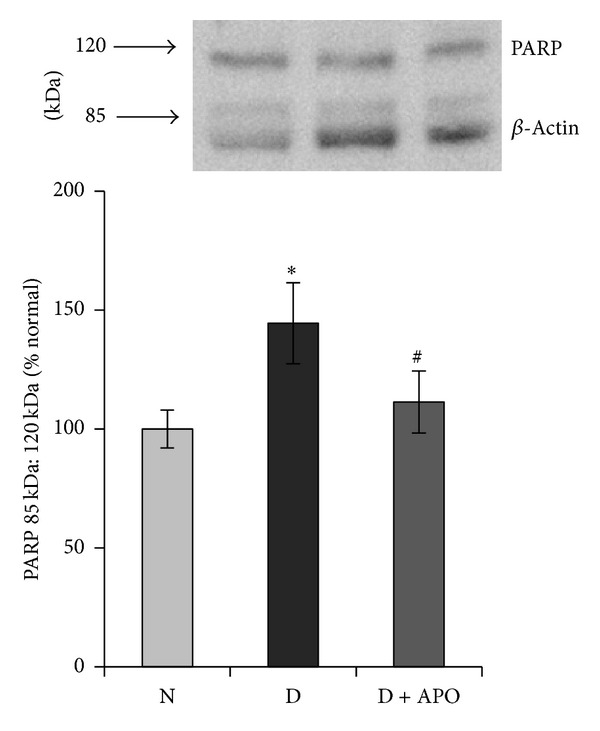
Diabetes-induced retinal PARP-1/2 activation were prevented by apocynin. PARP-1/2 expression in retina was quantified by Western blotting analysis using *β*-actin as a loading protein. Each measurement was made in duplicate or triplicate. The values are represented as mean ± SD of 5–7 rats in each of the three groups. N: normal, D: Diabetes, D + Apo: Diabetic rat received apocynin. *P < 0.05 compared to normal and ^#^ *P* < 0.05 compared to diabetes.

 Several previous studies have shown that PARP activation is mediated via excess ROS generation [31]. Therefore, we determined the expression levels PARP-1/2 expression in retina from the control, diabetic, and apocynin-treated rats. Pooled data accrued from multiple retinal preparations, as determined by Western blotting and densitometry, indicated a significant upregulation in the expression of PARP-1 in the diabetic retina compared with the control. Constant apocynin intake from the onset of diabetes significantly attenuated diabetes-induced upregulation of PARP-1/2 (Figure 8).

## 4. Discussion

In the present study, we investigated the role of PARP inhibition on diabetes-induced retinal neuropathy. We demonstrated that in the diabetic retina, PARP activation, ROS, p-ERK_1/2_, and cleaved caspase-3 levels were significantly increased. On the other hand, diabetes induced significant downregulation of the neurotrophin BDNF, the synaptic function marker synaptophysin, and GS. Administration of PARP inhibitor to diabetic rats significantly attenuated diabetes-induced increased expressions of PARP activation, ROS, p-ERK_1/2_, and cleaved caspase-3 and decreased BDNF, synaptophysin, and GS levels in the retinas.

Here, we report that retinal PARP immunoreactivity was upregulated in diabetic rats compared with nondiabetic controls. PARP immunoreactivity was localized primarily in the ganglion cell and inner nuclear layers of the retina. Similarly, the protein expressions of PARP-1/2 were upregulated by diabetes in the retina. Our results are consistent with previous reports that demonstrated upregulation of PARP expression in the diabetic retinas [28]. Here, we also report that diabetes-induced upregulation of PARP expression in the retina was prevented by the specific ROS inhibitor apocynin. Our results are also in agreement with previous reports that demonstrated increased levels of ROS and PARP in STZ-induced diabetic rat retinas [22, 23, 32]. Zheng et al. [33] reported that excessive oxidative stress is responsible for the upregulation of PARP in the diabetic retinas. Furthermore, supplementation of antioxidants such as simvastatin significantly attenuated diabetes-induced ROS generation, PARP activation, retinal capillary cells apoptosis, and formation of acellular capillaries in the retina. It was also shown that the early retinal changes induced by diabetes involve the overexpression of PARP, whose activation is associated with a retinal capillary cell apoptosis [22]. 

We showed here that the ROS level was reduced in the retinas of diabetic rats treated with the PARP inhibitor. Thus, our findings suggest that diabetes-induced ROS production in the retina appears to be also mediated by PARP. Our results are in agreement with previous reports that demonstrated that PARP inhibition attenuates the erectile impairment in diabetic rats by inhibition of ROS generation [34]. Similarly, Szabó et al. [35] suggested that ROS generation is a downstream target of hyperglycemia-induced PARP activation, as PARP inhibitors blocked the hyperglycemia-induced ROS generation in podocytes. Taken together, these findings suggest the presence of a positive feedback regulation between PARP activation and ROS generation in the diabetic retina.

The principal mediator responsible for the dysregulation of neurodegenerative markers such as BDNF, synaptophysin, and GS is ROS, which is produced by high glucose in the retina [36]. It has been proposed that diabetic-related retinal neuropathy is a result of impaired BDNF, synaptophysin, and GS expressions [3, 18, 26]. Previous studies demonstrated that one month of diabetes decreases the retinal expression of BDNF and synaptophysin [1, 3], and that the antioxidant lutein prevented BDNF and synaptophysin reduction and avoided increase in cleaved caspase-3 in the diabetic retina [3] suggesting that local oxidative stress has a neurodegenerative influence in diabetic retina. In addition, previous studies indicated that the expression of GS was significantly decreased in the diabetic rat retinas [17, 18]. Downregulation of GS activity resulted in elevated glutamate levels [17, 19, 20], which might induce retinal neuropathy via glutamate excitotoxicity. We found that PARP inhibition by 1,5-isoquinolinediol significantly prevented the diabetes-induced downregulation in BDNF, synaptophysin, and GS and upregulation of cleaved caspase-3 expressions. The mechanism by which the PARP inhibitor prevents diabetes-induced changes in the expression of BDNF, synaptophysin, GS, and caspase-3 in the retinas remains unclear. However, our findings suggested that inhibition of PARP that attenuates diabetes-induced changes in the expression of BDNF, synaptophysin, GS, and caspase-3 could be mediated by attenuating ROS generation. 

An additional mechanism that may contribute to the protective effect of PARP inhibition could be related to the suppression of ERK_1/2_ activation. ERK_1/2_ is a protein kinase intracellular signaling molecule that regulates the expression of genes involved in cell survival, apoptosis, and inflammatory response. Inhibition of ERK activation in the retina by its specific inhibitor U0126 blocks the actions of diabetes that causes upregulation of VEGF and inflammatory biomarkers, suggesting that ERK activation is involved in the pathogenesis of diabetic retinal dysfunction [37, 38]. Moreover, a previous study also documented that inhibition of ERK activation in the retina by lutein blocks the actions of diabetes that cause BDNF depletion and synaptophysin reduction [3]. It is known that the PARP activity is mediated by ERK-dependent phosphorylation [39–41] and persistent ERK activation has been shown in many complication-prone tissues in diabetes, including vascular smooth muscle cells, endothelial cells, peripheral nerves, and pericytes [42–44]. Here, we report that ERK_1/2_ is activated in the retinas of diabetic rats and 1,5-isoquinolinediol treatment inhibits ERK_1/2_ activation, thus suggesting that ERK activation may be a target of diabetes-associated PARP activation. Consistent with our findings, previous studies demonstrated that PARP-1 inhibition prevents ERK_1/2_ phosphorylation [45, 46], but the signaling events downstream of PARP-1 activation are not fully identified. The mechanism by which the PARP inhibitor prevents diabetes-induced ERK_1/2_ phosphorylation in the retinas could be by lowering ROS generation as ERK_1/2_ activation is known to be induced by oxidative stress [47–49].

In conclusion, these data suggest that PARP activation mediates diabetes-induced increased oxidative stress, downregulation of BDNF, synaptophysin, and GS, and upregulation of caspase-3. Collectively, our present data suggest that blocking PARP signaling pathways might be a novel therapeutic strategy for neuronal dysfunction in vision-threatening diabetic retinopathy.
